# Supplementary material for: Aβ1-42 and Tau as Potential Biomarkers for Diagnosis and Prognosis of Amyotrophic Lateral Sclerosis
Source: Int J Mol Sci. 2020 Apr 21;21(8):2911. doi: 10.3390/ijms21082911 (PMC7216266; doi:10.3390/ijms21082911)
Supplement: Supplementary file 1 [file ijms-21-02911-s001.pdf]

$A\beta$1-42 and Tau as potential biomarkers for diagnosis and prognosis of amyotrophic lateral sclerosis.

Débora Lanznaster, Rudolf C. Hergesheimer, Salah Eddine Bakkouche, Stephane Beltran, Patrick Vourc'h, Christian Andres, Diane Dufour-Rainfray, Philippe Corcia and Hélène Blasco.

Supplementary Table S1. Cox proportional hazards model for survival and biomarkers analyzed at baseline.

**Biomarker**	**HR**	**95% CI**	***p***
Aβ1-42	−0.0003	−0.001;0.0005	0.44
Phospho-Tau	−0.005	−0.02;0.007	0.42
Total Tau	−0.0002	−0.002;0.001	0.78
IATI	−0.09	−0.45;0.29	0.63
Ratio Aβ1-42/p-Tau	0.005	−0.02;0.03	0.67
p-tau/total Tau	−4.08	−12.21;3.81	0.31

HR: Hazar ratio; 95% CI: 95% confidence interval
